# Supplementary material for: The Hippo Pathway Effector YAP1 Regulates Intestinal Epithelial Cell Differentiation
Source: Cells. 2020 Aug 13;9(8):1895. doi: 10.3390/cells9081895 (PMC7463744; doi:10.3390/cells9081895)
Supplement: Supplementary file 1 [file cells-09-01895-s001.pdf]

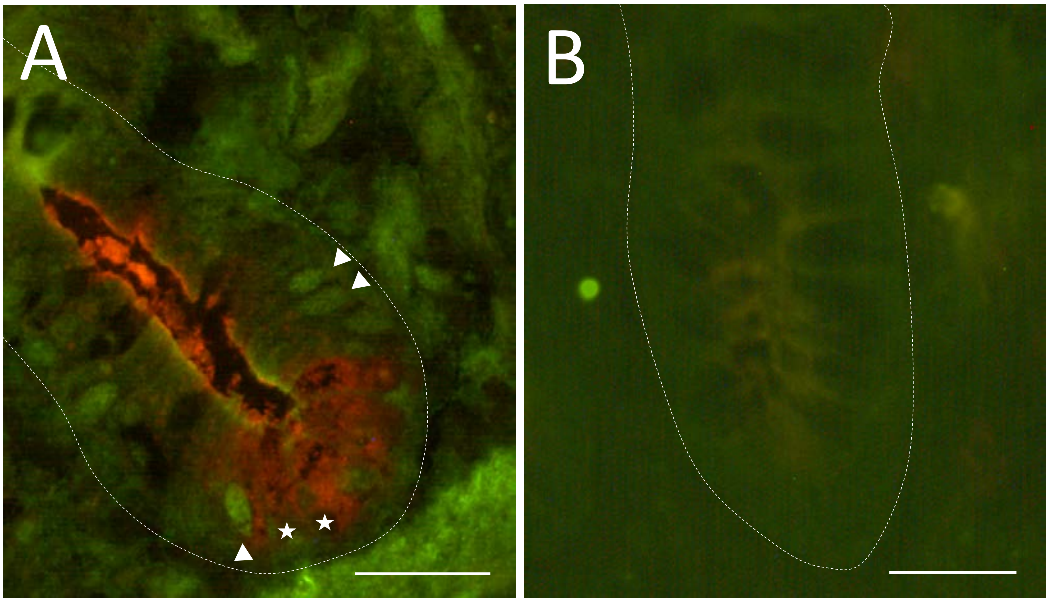


**Figure S1.** Nuclear localization of YAP1/TAZ in human intestinal crypt cells. **A**. Representative immunofluorescence staining for the detection of YAP1/TAZ (green) and DEFA5 (red) in the adult small intestine. Nuclear expression of YAP1/TAZ was observed in some of the cells mainly located at the base of the crypt (arrowheads) devoid of DEFA5 staining. **B**. Corresponding staining of a control section incubated unde the same conditions but without primary antibodies. Scale bars = 50 µm.

**Suppl Fig. 1**
